# Resistance of *Streptococcus suis* Isolates from the Czech Republic during 2018–2022

**DOI:** 10.3390/antibiotics11091214

**Published:** 2022-09-07

**Authors:** Katerina Nedbalcova, Ivana Kucharovicova, Monika Zouharova, Katarina Matiaskova, Natalie Kralova, Marek Brychta, Bronislav Simek, Tomas Pecha, Hana Plodkova, Jan Matiasovic

**Affiliations:** 1 Veterinary Research Institute, Hudcova 296/70, 621 00 Brno, Czech Republic; 2 State Veterinary Institute, Rantirovska 93, 586 05 Jihlava, Czech Republic; 3 Department of Experimental Biology, Faculty of Science, Masaryk University, Kamenice 753/5, 625 00 Brno, Czech Republic

**Keywords:** antimicrobial susceptibility testing, serotypization, minimal inhibitory concentration, infectious disease, pigs

## Abstract

A determination of susceptibility/resistance to antimicrobials via serotype was carried out in 506 field isolates of *Streptococcus suis*, originating from pig farms in the Czech Republic in the period 2018–2022. A very high level of susceptibility of *S. suis* isolates was found to amoxicillin, in combination with clavulanic acid and sulfamethoxazole potentiated with trimethoprim. None of the tested isolates were resistant to these antimicrobial substances. Only two isolates were found to be intermediately resistant to enrofloxacin in 2020. With regard to ceftiofur, one isolate was intermediately resistant in 2020 and 2022, and two isolates were intermediately resistant in 2018 and 2021. A low level of resistance was detected to ampicillin (0.6% in 2021) and to florfenicol (1.15% in 2019; 1.3% in 2022). With regard to penicillin, a medium level of resistance was detected in 2018 (10.6%), but a low level of resistance was found in the following years (7.0% in 2019; 3.1% in 2020; 3.3% in 2021; 3.9% in 2022). On the contrary, a high or very high level of resistance was found to tetracycline (66.0% in 2018; 65.1% in 2019; 44.35% in 2020; 46.4% in 2021; 54.0% in 2022). Using molecular and serological methods, serotype 7 (16.4%) was determined to be predominant among *S. suis* isolates, followed by serotypes 1/2, 2, 9, 4, 3, 1, 29, 16, and 31 (10.7%; 8.5%; 5.7%; 5.5%; 4.5%; 4.3%; 3.6%; 3.4%; 3.4%, respectively). Other serotypes were identified among the investigated strains either rarely (up to 10 cases) or not at all. A relatively high percentage of isolates were detected as non-typeable (79 isolates; 15.6%). Dependence of resistance upon serotype assignment could not be proven in all but serotype 31, wherein all isolates (*n* = 17) were resistant or intermediately resistant to clindamycin, tilmycosin, tulathromycin, and tetracycline. The resistance to clindamycin and tetracycline may be related to the high consumption of these antibiotics on pig farms at present or in previous years. Macrolides (tilmicosin and tulathromycin) and tiamulin are not suitable for the treatment of streptococcal infections, but are used on pig farms to treat respiratory infections caused by gram-negative bacteria, so they were included in the study.

## 1. Introduction

Diseases caused by *Streptococcus suis* are currently a major economic problem on pig farms worldwide [1]. Although *S. suis* is primarily considered to be the causative agent of pig infections, it is increasingly being identified as the zoonotic agent responsible for serious human infections. However, human cases of infection are rather sporadic and are usually caused by the transmission of *S. suis* from contaminated animals or pork products to humans via skin lesions or the oral route [2].

Meningitis and septicemia are considered the most common and significant clinical manifestations of the disease in pigs, but *S. suis* can also cause endocarditis, pneumonia, arthritis, polyserositis, and vaginitis [3].

Antimicrobials are used to treat *S. suis* infections. However, the inappropriate or careless use of antimicrobials to treat infections in human and veterinary medicine has led to antimicrobial resistance (AMR), which has become a global problem in recent years [4,5,6]. AMR significantly increases the risk of therapeutic failure. Due to the increase in AMR in recent decades, the susceptibility of bacterial pathogens to antimicrobials should be carefully monitored to ensure the long-term efficacy of authorized antibacterial drugs. AMR monitoring is currently supported by many national and international policy agendas. For the monitoring of AMR, the determination of the minimum inhibitory concentrations (MICs) of antimicrobials is considered the gold standard method [7].

The strains of *S. suis* are not antigenically uniform. Based on the diversity of capsular polysaccharides (CPSs), 35 different serotypes (1–34 and 1/2) [8,9,10,11] have been described. Some serotypes have also been classified as other bacterial species based on their genomic analysis [12,13]. Therefore, we currently only know of 29 *S. suis* serotypes. The original serotypes 20, 22, 26, 32, 33, and 34 are sometimes referred to as *S. suis*-like strains [14].

The serological methods—agglutination or coagglutination tests using serotype-specific antisera—are still considered the gold standard for serotyping, and they were originally used for *S. suis* serotyping [15]. Over time, PCR methods based on the detection of genes encoding the CPS production of individual serotypes have been developed to facilitate the differentiation of *S. suis* serotypes [14,16]. However, serotypes 2 and 1/2, like serotypes 1 and 14, cannot be clearly distinguished from each other because they cross-react with each other during the serological determination, or when performing specific PCRs to detect genes encoding the CPS production of individual serotypes [14,16,17]. This is very important because serotypes 2 and 14 are associated with human diseases [18]. To date, whole genome sequencing has been the only available method that can distinguish these cross-reacting serotypes due to the presence of single nucleotide polymorphism in the cpsK gene [19]. However, this method is not suitable for routine testing because it is equipment-intensive and quite expensive. Recently, a simple and rapid PCR-RFLP method for differentiation between the *S. suis* serotype 2 from serotype 1/2 and serotype 1 from serotype 14 was developed, which is also suitable for routine diagnostics [20].

This study summarizes the results of AMR monitoring of *S. suis* isolates originating from sick pigs on farms in the Czech Republic during the period 2018–2022. It also contains information on the occurrence of resistance to monitored antimicrobials depending on the isolates belonging to individual *S. suis* serotypes.

## 2. Results

**Figure 1:**
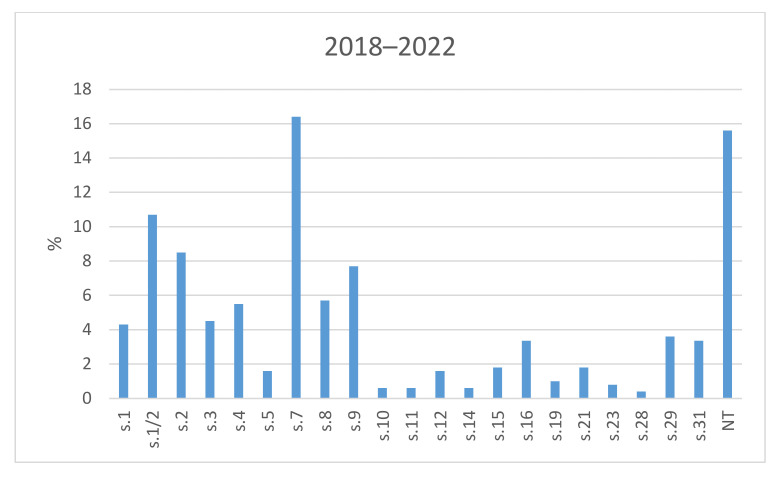
The prevalence of serotypes of *S. suis* isolates from Czech farms in 2018–2022. NT = Non-typeable isolates.

The results of the serotyping of all tested field isolates of *S. suis* from Czech farms during 2018–2022 are shown in Figure 1. Of all of the 506 isolates, serotype 7 was detected most often—in 83 isolates (16.4%). In total, 54 isolates (10.7%) were determined to be serotype 1/2, 43 isolates (8.5%) were found to be serotype 2, and serotype 9 was identified in 39 isolates (7.7%). Several isolates were determined as serotype 8, 4, 3, 1, 29, 16, and 31 (29 isolates—5.7%; 28 isolates—5.5%; 23 isolates—4.5%; 22 isolates—4.3%; 18 isolates—3.6%; 17 isolates—3.4%; 17 isolates—3.4%). Other serotypes were identified among the investigated strains either rarely (up to 10 cases) or not at all. A relatively high percentage of isolates were detected as non-typeable (79 isolates; 15.6%).

**Figure 2:**
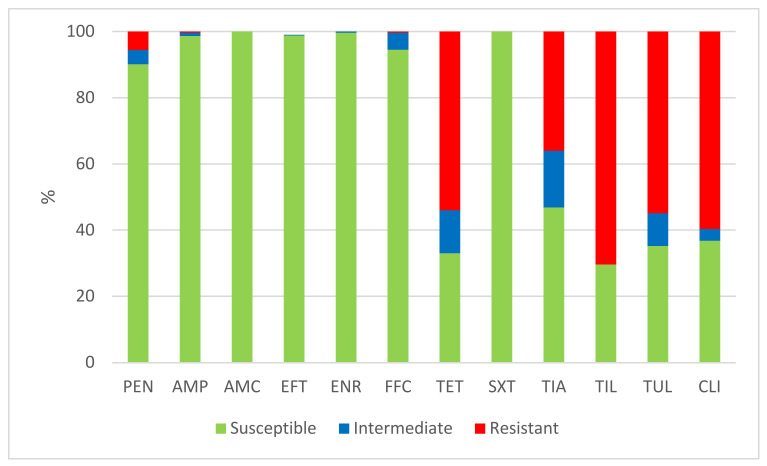
The percentage of susceptible, intermediately resistant, and resistant *S. suis* isolates from Czech farms in 2018–2022 (*n* = 506). PEN = Penicillin; AMP = Ampicillin; AMC = Amoxicillin/Clavulanate 2/1; EFT = Ceftiofur; ENR = Enrofloxacin; FFC = Florfenicol; TET = Tetracycline; STX = Trimethoprin/Sulfamethoxazole 1/19; TIA = Tiamulin; TIL = Tilmicosin; TUL = Tulathromycin; CLI = Clindamycin.

**Figure 3:**
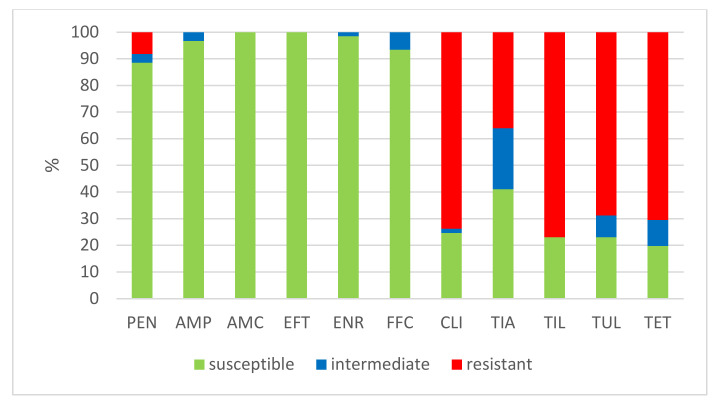
The percentage of susceptible, intermediately resistant, and resistant *S. suis* isolates from nasal swabs and tonsils. (*n* = 61). PEN = Penicillin; AMP = Ampicillin; AMC = Amoxicillin/Clavulanate 2/1; EFT = Ceftiofur; ENR = Enrofloxacin; FFC = Florfenicol; TET = Tetracycline; STX = Trimethoprin/Sulfamethoxazole 1/19; TIA = Tiamulin; TIL = Tilmicosin; TUL = Tulathromycin; CLI = Clindamycin.

**Figure 4:**
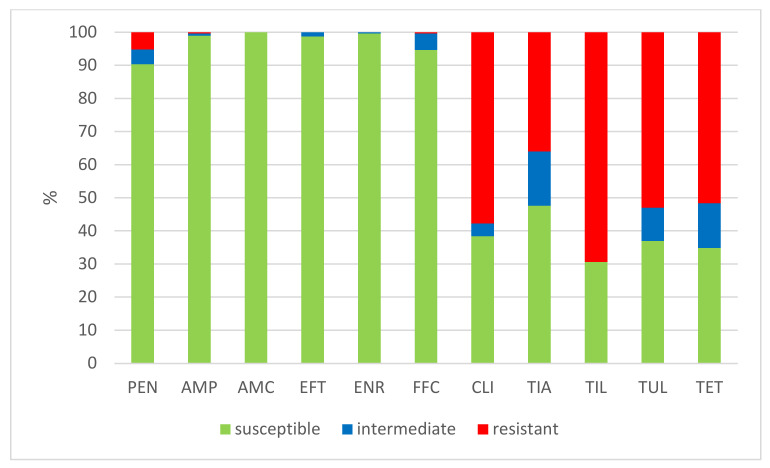
The percentage of susceptible, intermediately resistant, and resistant *S. suis* isolates from systemic organs in 2018–2022 (*n* = 445). PEN = Penicillin; AMP = Ampicillin; AMC = Amoxicillin/Clavulanate 2/1; EFT = Ceftiofur; ENR = Enrofloxacin; FFC = Florfenicol; TET = Tetracycline; STX = Trimethoprin/Sulfamethoxazole 1/19; TIA = Tiamulin; TIL = Tilmicosin; TUL = Tulathromycin; CLI = Clindamycin.

The results of the antimicrobial susceptibility testing of all 506 *S. suis* isolates are summarized in Table 1. The MICs distribution for antimicrobials (the number of detected isolates with the corresponding value of MIC given in the table header), as well as the percentages of susceptible, intermediately resistant, and resistant isolates, and the MIC_50_ and MIC_90_ values, in individual years and overall, are listed in Table 1. The average percentages of susceptible, intermediately resistant, and resistant *S. suis* isolates of the 506 tested isolates are given in Figure 2. The average percentages of susceptible, intermediately resistant, and resistant *S. suis* isolates from the upper respiratory tract (nasal swabs and tonsils) are given in Figure 3, and the average percentages of susceptible, intermediately resistant, and resistant *S. suis* isolates from systemic organs are given in Figure 4.

A very high level of susceptibility in the *S. suis* isolates was found to amoxicillin, in combination with clavulanic acid and sulfamethoxazole potentiated with trimethoprim. None of the tested isolates were resistant to these antimicrobial substances. Only one or two isolates were found to be intermediately resistant to ceftiofur and enrofloxacin in individual years (see Table 1). A low level of resistance was detected to ampicillin (0.6% in 2021) and to florfenicol (1.15% in 2019; 1.3% in 2022). Somewhat more isolates intermediately resistant to florfenicol were detected (3.2% in 2018; 1.15% in 2019; 5.2% in 2020; 11.1% in 2021). With regard to penicillin, a mediate level of resistance was detected in 2018 (10.6%), but a low level of resistance was found in the following years (7.0% in 2019; 3.1% in 2020; 3.3% in 2021; 3.9% in 2022). On the other hand, high or very high levels of resistance were found to tetracycline (66.0% in 2018; 65.1% in 2019; 44.35% in 2020; 46.4% in 2021; 54.0% in 2022), tiamulin (38.3% in 2018; 32.6% in 2019; 28.8% in 2020; 34.6% in 2021; 48.7% in 2022), tilmicosin (68.0% in 2018; 62.8% in 2019; 63.9% in 2020; 81.0% in 2021; 69.7% in 2022), tulathromycin (52.1% in 2018; 53.6% in 2020; 69.9% in 2021; 54.0% in 2022), and clindamycin (40.4% in 2018; 59.3% in 2019; 58.8% in 2020; 68.6% in 2021; 67.1% in 2022).

**Figure 5:**
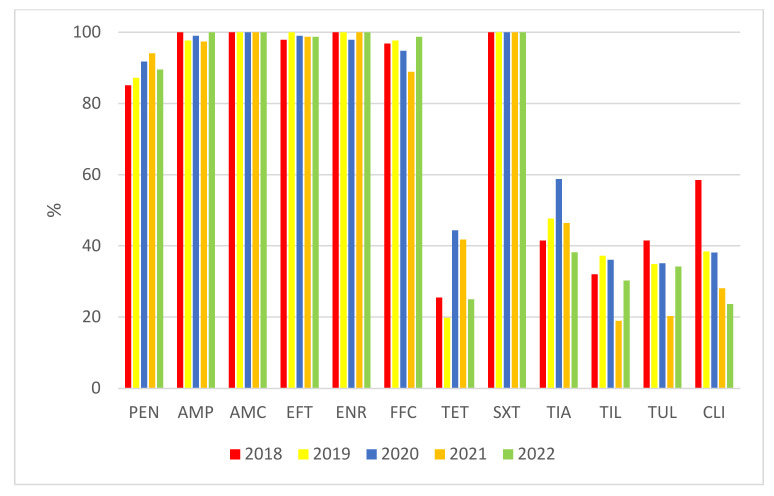
Comparison of the percentages of isolates of S. suis susceptible to the tested antimicrobials in individual years. PEN = Penicillin; AMP = Ampicillin; AMC = Amoxicillin/Clavulanate 2/1; EFT = Ceftiofur; ENR = Enrofloxacin; FFC = Florfenicol; TET = Tetracycline; STX = Trimethoprin/Sulfamethoxazole 1/19; TIA = Tiamulin; TIL = Tilmicosin; TUL = Tulathromycin; CLI = Clindamycin.

When we compare the percentages of susceptible isolates in the individual monitored years, we find noticeable differences between antimicrobials, finding more isolates (penicillin and especially tetracycline, tiamulin, tilmicosin, tulathromycin, and clindamycin) that are resistant to them (Figure 5).

**Figure 6:**
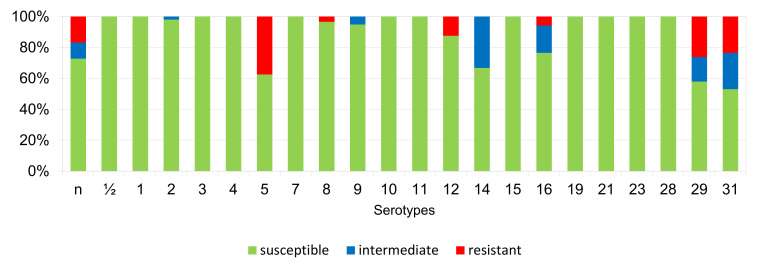
Percentage representation of isolates that are resistant, intermediately resistant, and susceptible to penicillin belonging to individual *S. suis* serotypes from the Czech Republic during 2018–2022 (*n* = 506). *n* = non-typeable isolates.

**Figure 7:**
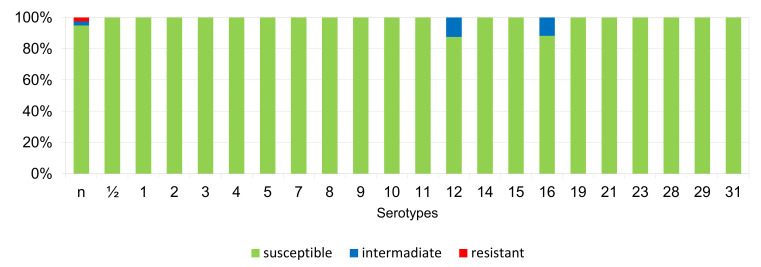
Percentage representation of isolates resistant, intermediately resistant, and susceptible to ampicillin belonging to individual *S. suis* serotypes from the Czech Republic during 2018–2022 (*n* = 506). *n* = non-typeable isolates.

**Figure 8:**
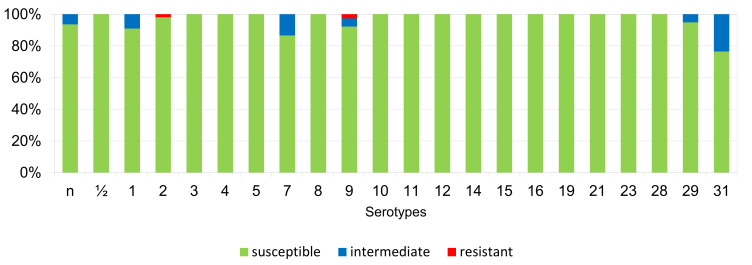
Percentage representation of isolates resistant, intermediately resistant, and susceptible to florfenicol belonging to individual *S. suis* serotypes from the Czech Republic during 2018–2022 (*n* = 506). *n* = non-typeable isolates.

**Figure 9:**
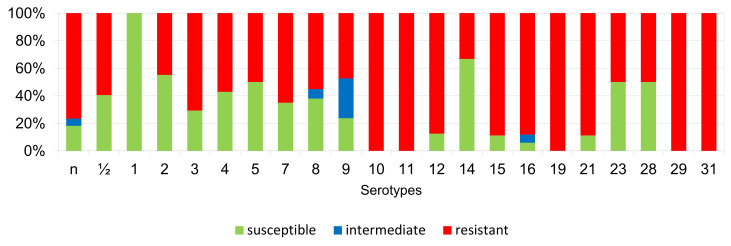
Percentage representation of isolates resistant, intermediately resistant, and susceptible to clindamycin belonging to individual *S. suis* serotypes from the Czech Republic during 2018–2022 (*n* = 506). *n* = non-typeable isolates.

**Figure 10:**
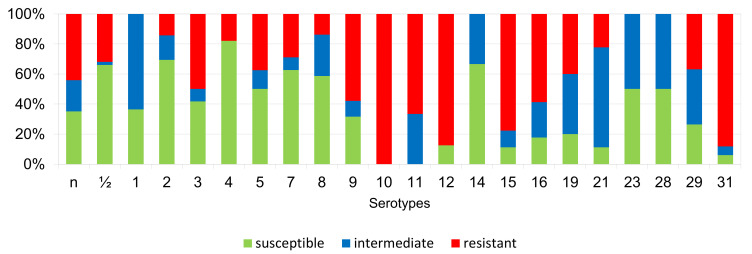
Percentage representation of isolates resistant, intermediately resistant, and susceptible to tiamulin belonging to individual *S. suis* serotypes from the Czech Republic during 2018–2022 (*n* = 506). *n* = non-typeable isolates.

**Figure 11:**
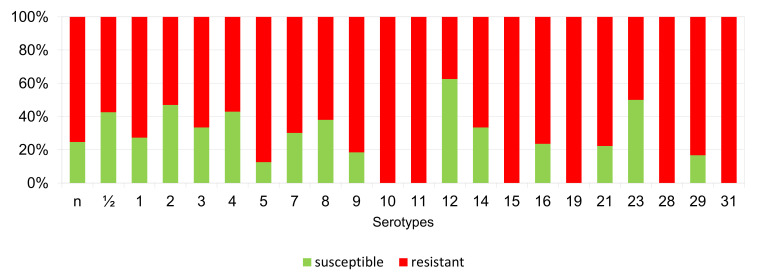
Percentage representation of isolates resistant, intermediately resistant, and susceptible to tilmicosin belonging to individual *S. suis* serotypes from the Czech Republic during 2018–2022. (*n* = 506). *n* = non-typeable isolates.

**Figure 12:**
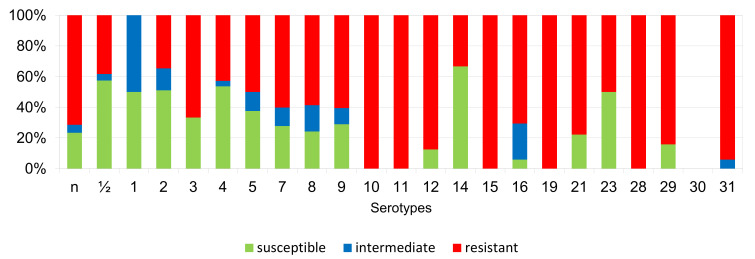
Percentage representation of isolates resistant, intermediately resistant, and susceptible to tulathromycin belonging to individual *S. suis* serotypes from the Czech Republic during 2018–2022 (*n* = 506). *n* = non-typeable isolates.

**Figure 13:**
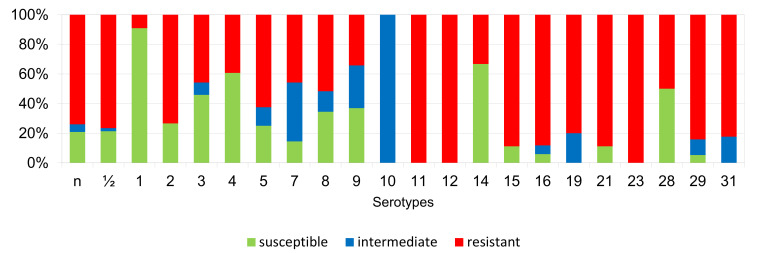
Percentage representation of isolates resistant, intermediately resistant, and susceptible to tetracycline belonging to individual *S. suis* serotypes from the Czech Republic during 2018–2022 (*n* = 506).

The percentages of resistant, intermediately resistant, and susceptible isolates belonging to individual serotypes in which we detected resistant *S. suis* isolates (penicillin, ampicillin, florfenicol, clindamycin, tiamulin, tilmicosin, tulathromycine, and tetracycline) are shown in Figure 6, Figure 7, Figure 8, Figure 9, Figure 10, Figure 11, Figure 12 and Figure 13.

According to Figure 6, Figure 7, Figure 8, Figure 9, Figure 10, Figure 11, Figure 12 and Figure 13, we can generally summarize that during the monitoring of resistance in this study, the dependence of the frequency of occurrence of resistant isolates on the serotype the isolates belonged to was not proven. However, it can be seen that, with regard to clindamycin, tiamulin, tilmicosin, tulathromycin and tetracycline, a high detection rate of intermediately resistant or resistant strains was recorded in serotypes 10–31, with the exception of serotypes 14, 23, and 28. All isolates of serotype 31 (*n* = 17) were intermediately resistant or resistant to clindamycin, tilmicosin, tulathromycin, and tetracycline. Although present in low numbers (*n* lower than 10 isolates per serotype), all isolates of serotypes 10, 11, and 19 were also intermediately resistant or resistant to clindamycin, tilmicosin, tulathromycin, and tetracycline, and all serotype 10 isolates were resistant to tiamulin. *n* = non-typeable isolates.

## 3. Discussion

Knowledge of the resistance of pathogens present in the population is critically important for making informed decisions about which antimicrobial to choose in field conditions, if its use is necessary. Here, we present the results of a project focused on the description of *S. suis* isolates collected between the 2018 and 2022 in the Czech Republic. With respect to the frequent import and export of pigs between countries of the European Union, we believe our findings can be informative for neighboring countries.

In this study, the resistance to common antimicrobials used for the treatment of *S. suis* infections was tested in 506 isolates. All isolates were identified by MALDI-TOF; the presence of the *recN* gene was confirmed by PCR, and the isolates were serotyped. The resistance to the commonly used antimicrobials of tetracycline, tiamulin, tilmicosin, tulathromycin, and clindamycin was high. The high frequencies of resistance to tetracycline correlate with the frequent use of this antibiotic for the treatment *S. suis* infections on pig farms [21,22]. Moreover, high levels of occurrence of resistance have also been described in protein synthesis inhibitors, such as tetracycline and clindamycin [4,23,24]. Resistance to tiamulin and macrolides (tilmicosin and tulathromycin) is reported very often in the antimicrobial susceptibility testing of *Streptococcus* spp. These antimicrobials are thus not suitable for use in the treatment of streptococcal infection. We tested them due to their common use on pig farms against outbreaks of other diseases caused by Gram-negative bacteria, mainly members of the families *Pasteurellaceae*–*Actinobacillus pleuromoniae*, *Pasteurella multocida*, or *Glaesserella parasuis* [25]. The high degree of resistance to these antimicrobials in *S. suis* isolates may be due to the horizontal transfer of resistance genes between different bacterial populations [26,27].

Beta-lactam antibiotics are often used for *S. suis* infection treatment. In the past, the first choice to control the occurrence and spread of *S. suis* was feeding mixtures supplemented with beta-lactams, such as penicillin or amoxicillin [28]. Nowadays, due to the presence of bacterial populations resistant, or even multi-resistant, to antibiotics, this usage must be well justified [29].

If we compare the results of published studies dealing with monitoring the resistance of *S. suis* isolates from around the world, we find a high level of resistance to tetracyclines, clindamycin, tiamulin, and macrolides, and, on the other hand, a relatively small level of isolates resistant to penicillin antibiotics or fluoroquinolones [28]. In Spain, very low resistance to beta-lactam antibiotics and fluoroquinolones was found in *S. suis* isolates, but resistance to tetracyclines using current breakpoints exceeded 97% [21]. In another study, summarizing the results of antimicrobial resistance surveillance from seven EU countries, a high percentage of isolates of *S. suis* were susceptible to ceftiofur, cefquinome, and penicillin. A very high level of isolates was resistant to tetracycline (80%). Enrofloxacin and florfenicol, similarly to our study, were described with moderate activity (MIC_90_ values of 0.5 and 2 mg/L, close to the susceptibility breakpoints) in 2006, but none of the isolates were resistant to these antibiotics [30]. In the Netherlands, all *S. suis* isolates (*n* = 848) were susceptible to ampicillin, while resistance to penicillin was 3%. Resistance to tetracycline was at the level of 81%. Similar results have been presented by the VetPath study (European monitoring of AMR in veterinary pathogens) [7,31,32]. Surprisingly, a high level of resistance to trimethoprim/sulfamethoxazole was reported in Denmark, because trimethoprim/sulfamethoxazole was presented as effective, with sensitivity to more than 90% of the isolates tested in other studies [33]. In the results of AMR monitoring in Sweden, China, and New Zealand published this year, a high percentage of *S. suis* isolates especially resistant to tetracycline (over 90%) was presented, as was a very good sensitivity of *S. suis* to beta-lactams and fluoroquinolones [1,34,35].

In an attempt to understand resistance among different serotypes, the serotyping of *S. suis* isolates was performed based on CPS differences [16,19,20,36,37]. A relatively high percentage of isolates were marked as non-typeable. Mutations during subcultivation [16] can lead to the loss of the isolate’s ability to produce a capsule, and these isolates are serologically non-typeable [38].

According to published studies, the occurrence of individual serotypes in the monitored geographic areas varies, and often changes over time [24]. Serotype 2 was presented as predominant worldwide (in North America, Europe, and Asia) in recent years [4,39,40,41]. In our study, we detected serotype 7 (16.4%) as the most predominant, while an even higher incidence was reported in North America and Thailand [40,41,42]. Based on a new differentiation method [20], only 8.5% of our isolates were determined as serotype 2, but on the other hand, we identified 10.7% of isolates as serotype 1/2, which is a total of 19.2%. Until recently, no methods have been available to unequivocally distinguish these two cross-reacting serotypes from each other, it is possible that a higher incidence of serotype 2 may have been described in previously published studies at the expense of serotype 1/2. Overall, we found that most of our isolates were non-typeable, or were serotypes 1–9, including serotype 1/2. The presence of susceptible, intermediately resistant, or resistant strains was found within these isolates. On the other hand, within serotypes 10–31 (excluding serotypes 14, 23, and 28) were isolates mostly resistant, or intermediately so, to clindamycin, tilmicosin, tulathromycin, and tetracycline. However, these isolates were present in low numbers, and this finding may be the result of chance. On the other hand, this may not be the case for serotype 31, wherein all isolates (*n* = 17) were intermediately resistant or resistant to clindamycin, tilmicosin, tulathromycin, and tetracycline.

## 4. Materials and Methods

### 4.1. Isolates

A total of 506 *S. suis* isolates were obtained from the systemic organs of dead pigs or from nasal swabs of diseased pigs on Czech farms during 2018–2022 (94 isolates in 2018; 86 isolates in 2019; 97 isolates in 2020; 153 isolates in 2021; 76 isolates in 2022). The origin of the isolates is specified in Table 2. *S. suis* was either the primary pathogen or part of the multifactorial infectious disease with respiratory symptoms in the pigs. Each of the tested isolates was derived from a different animal.

The *S. suis* isolates were obtained from samples via commonly available basic bacteriological culture procedures and methods—swabs and clinical and sectional material were cultured on blood agar (Blood Agar Base No.2 (Oxoid, Basingstoke, UK)) with 5% defibrinated ram blood (LabMediaServis s.r.o., Jaroměř, Czech Republic). The usual incubation time of primocultures is 22–48 h, at 37 ± 1 °C. Suspected colonies of *S. suis* were isolated on blood agar, and for better and faster growth the isolate was cultured in a microaerophilic atmosphere (CampyGen, Oxoid, Basingstoke, UK) for 22–24 h at 37 °C. After obtaining a pure bacterial culture, the pathogen was identified by standard operating procedures, namely the MALDI-TOF method (Matrix Assisted Laser Desorption/Ionization–Time of Flight) using a Bruker Microflex mass spectrometer including Maldi Biotyper 3.0 software, Database CD BTYP3.0 Library (updated version). The database was used to obtain rapid results regarding the genus and species of the bacterium. Furthermore, a standard biochemical examination was performed with a commercial STREPTOTEST 24 (Erba Lachema, Brno, Czech Republic) and a microscopic examination (Gram staining, Merck, Rahway, NJ, USA). Simultaneously and for completeness, the rapid slide latex agglutination of all isolates was performed with a commercial DiaMondiaL Strep Kit (Biomedica CS, Brno, Czech Republic).

### 4.2. Serotyping

For serotype determination, multiplex PCR in four separate PCR reactions was carried out according to the method described previously [36], with some modifications. The primers targeted the following genes: (i) glycosyltransferase genes *cps1J*, *cps14J*, *cps1/2J*, *cps2J*, *cps3J*, *cps7H*, *cps9H*, *cps16K*, *cps21N*, *cps23I* and *cps24L*; (ii) capsular polysaccharide repeat unit transporter genes *cps3K*, *cps4M* and *cps5N*; (iii) UDP-glucose dehydrogenase gene *cps4N*; (iv) oligosaccharide repeat unit polymerase genes *cps6I*, *cps10M*, *cps11N*, *cps12J*, *cps13L*, *cps15K*, *cps17O*, *cps18N*, *cps19L*, *cps25M*, *cps27K*, *cps28L*, *cps29L*, *cps30I* and *cps31L*; (v) N-acetylmannosaminyltransferase gene *cps8H*; and (vi) glycerophosphotransferase gene *cps25N*.

The first PCR reaction included the primers for serotypes 1 + 14, 2 + 1/2, 3, 7, 9, 11, 14, and 16, and species-specific gene *recN*. This *recN* gene was added for *S. suis* verification. It is specific to *S. suis* and it is not amplified in certain serotypes (20, 22, 26, 32, 33, and 34), which have recently been excluded from the *S. suis* species after reclassification [37]. The second reaction included the primers for serotypes 4, 5, 8, 12, 18, 19, 24, and 25; the third included the primers for serotypes 6, 10, 13, 15, 17, 23, and 31; and the fourth reaction included the primers for serotypes 21, 27, 28, 29, and 30. Serotypes identified as 1 or 14 and 2 or 1/2 were further distinguished by the PCR-RFLP method detecting polymorphism in the *cpsK* gene [20].

Strains not-typeable by PCR were serotyped by a co-agglutination test. Antisera against all the reference strains were prepared in rabbits, and co-agglutination reagents were prepared according to the previously described coagglutination test [43]. No positive reactions were obtained.

### 4.3. Antimicrobial Susceptibility Testing

The antimicrobial susceptibility testing of six selected antimicrobials and two combinations of antimicrobials was performed by determining the minimum inhibitory concentrations (MICs) using the microdilution method. Based on clinical breakpoints, the following classification of isolates into sensitivity categories (susceptible, intermediately resistant, resistant) was performed, according to internationally recognized methodology accredited by the Clinical and Laboratory Standards Institute [44], with the exception of tiamulin, for which the interpretative criteria suggested in previously published study were used [45]. However, we derived criteria for *Pasteurella multocida* (tilmicosin and tulathromycin) and for human (clindamycin) antimicrobials from CLSI [44], but this offered no interpretative criteria for *Streptococcus* spp. MICs were determined using diagnostic sets made at the Veterinary Research Institute in Brno, Czech Republic (Table 3). The quality control of the MIC determination was assessed by parallel examination of the control reference strain *Streptococcus pneumoniae* ATCC 49619 [44,46].

The MIC_50_ and MIC_90_ values were determined from cumulative results regarding the lowest antimicrobial concentration in mg/L that inhibits the growth of 50% and 90% of isolates [47]. The level of resistance was assessed according to following scale: rare (<0.1%), very low (0.1–1%), low (>1–10%), medium (>10–20%), high (>20–50%), very high (>50–70%) and extremely high (>70%).

## 5. Conclusions

Pathogenic strains of *S. suis* often cause serious neurological and systemic diseases in pigs. For the treatment of these infections, the precise identification of the causative agent of the disease and the use of correctly selected antimicrobials are key. There is still little information available about the population structure of *S. suis* in the Czech Republic. Thus, this study presents one of the first overviews of the antimicrobial susceptibility testing of *S. suis* field isolates in the Czech Republic.

## Figures and Tables

**Table 1:** MICs distribution for antimicrobials; percentages of susceptible, intermediately resistant, and resistant isolates, and MIC_50_ and MIC_90_ values in *S. suis* isolates from Czech farms in 2018–2022.

	MIC (mg/L)														S (%)	I (%)	R (%)	MIC_50_ (mg/L)	MIC_90_ (mg/L)
	0.03	0.06	0.125	0.25	0.5	1	2	4	8	16	32	64	128	256					
**2018: *n* = 94**																			
PEN	27	34	10	9	4	7	3								85.1	4.3	10.6	0.06	1
AMP	43	30	16	5											100	0	0	0.06	0.125
AMC				93	1										100	0	0	≤0.25	≤0.25
EFT			37	25	9	13	8	2							97.9	2.1	0	0.25	2
ENR		5	9	49	31										100	0	0	0.25	0.5
FFC					1	16	74	3							96.8	3.2	0	2	2
TET				20	4	8	4	4	5	7	37	5			25.5	8.5	66	16	32
SXT		66	17	9	2										100	0	0	≤0.06	0.25
TIA				2	5	6	5	21	12	7	9	27			41.5	21.2	38.3	8	>32
TIL								2	9	20	22	6	10	25	32	-	68	64	>128
TUL							3	10	14	12	6	5	1	43	41.5	6.4	52.1	64	>128
CLI			46	9	1	1	1	5	4	3	24				58.5	1.1	40.4	0.25	>16
**2019: *n* = 86**																			
PEN	30	34	4	7	4	2	4	1							87.2	4	7	0.06	0.5
AMP	37	28	16	1	2	2									97.7	2.3	0	0.06	0.125
AMC				85	1										100	0	0	≤0.25	≤0.25
EFT			34	32	7	1									100	0	0	0.25	1
ENR		3	25	45	13										100	0	0	0.25	0.5
FFC					1	20	63	1	1						97.7	1.15	1.15	2	2
TET				11	6	13	1		5	14	34	2			19.8	15.1	65.1	16	32
SXT		61	15	8	2										100	0	0	≤0.06	0.25
TIA				2	6	2	10	21	11	6	5	23			47.7	19.7	32.6	8	>32
TIL						1			2	29	14		1	39	37.2	-	62.8	32	>128
TUL						1	2	3	13	11	9	6	2	39	34.9	10.4	54.7	64	>128
CLI			26	7	2	2		8	3	1	37				38.4	2.3	59.3	4	>16
**2020: *n* = 97**																			
PEN	43	37	7	2	5	1	2								91.8	5.1	3.1	0.06	0.25
AMP	52	37	2	4	1		1								99	1	0	≤0.03	0.125
AMC				95	2										100	0	0	≤0.25	≤0.25
EFT			63	22	4	5	2	1							99	1	0	≤0.125	0.5
ENR		2	16	59	18	2									97.9	2.1	0	0.25	0.5
FFC					1	25	66	5							94.8	5.2	0	2	2
TET				35	8	11	6	1	4	9	18	5			44.35	11.3	44.35	1	32
SXT		78	12	6	1										100	0	0	≤0.06	0.125
TIA					11	6	9	27	7	5	12	16			58.8	12.4	28.8	4	>32
TIL									6	29	20			42	36.1	-	63.9	128	>128
TUL						1	1	5	9	18	11	10	1	41	35.1	11.3	53.6	64	>128
CLI			33	4	3		1	6	1	5	44				38.1	3.1	58.8	16	>16
**2021 (*n* = 153)**																			
PEN	79	59	4	2	4	2	1	2							94.1	2.6	3.3	≤0.03	0.06
AMP	84	57	4	3	1	3	1								97.4	2	0.6	≤0.03	0.06
AMC				151	2										100	0	0	≤0.25	≤0.25
EFT			111	23	6	6	5	2							98.7	1.3	0	≤0.125	0.5
ENR		2	37	77	37										100	0	0	0.25	0.5
FFC						42	94	17							88.9	11.1	0	2	4
TET				52	12	18	11		1	11	35	13			41.8	11.8	46.4	1	32
SXT		123	19	6	3	2									100	0	0	≤0.06	0.125
TIA				5	16	10	17	23	15	14	12	41			46.4	19	34.6	8	>32
TIL							4	8	1	16	37	2	2	83	19	-	81	>128	>128
TUL						10	4	3	8	6	15	21	4	82	20.3	9.8	69.9	>128	>128
CLI			35	8	5	1	2	12	7	1	82				28.1	3.3	68.6	>16	>16
**2022 (*n* = 76)**																			
PEN	32	26	5	5	5	2	1								89.5	6.6	3.9	0.06	0.5
AMP	36	29	4	6	1										100	0	0.4	0.06	0.125
AMC				76											100	0	0	≤0.25	≤0.25
EFT			57	6	7	3	2	1							98.7	1.3	0	≤0.125	0.5
ENR		1	8	49	18										100	0	0	0.25	0.5
FFC						14	61			1					98.7	0	1.3	2	2
TET				14	5	16	2		2	7	28	2			25	21	54	8	32
SXT		61	10	3	1	1									100	0	0	≤0.06	0.125
TIA					2	3	7	17	4	6	9	28			38.2	13.1	48.7	16	>32
TIL								1	1	21	21			32	30.3	-	69.7	>128	>128
TUL							5	9	2	10	9	8	1	32	34.2	11.8	54	>128	>128
CLI			17	1	7	1		10	4	2	34				23.7	9.2	67.1	>16	>16
**2018–2022 (*n* = 506)**																			
PEN	211	190	30	25	22	14	11	3							90.1	4.3	5.6	0.06	0.25
AMP	252	181	42	19	5	5	2								98.6	1	0.4	0.06	0.125
AMC				500	6										100	0	0	≤0.25	≤0.25
EFT			302	108	33	40	17	6							98.8	0.2	0	≤0.125	1
ENR		13	95	279	117	2									99.6	0.4	0	0.25	0.5
FFC					3	117	358	26		2					94.5	5.1	0.4	2	2
TET				132	35	66	24	5	17	48	152	27			33	13	54	2	32
SXT		389	73	32	9	3									100	0	0	≤0.06	0.125
TIA				13	40	27	48	109	49	38	47	135			46.8	17.2	36	8	>32
TIL						1	4	11	19	115	114	8	13	221	29.6	-	70.4	32	>128
TUL						12	15	30	46	57	50	50	9	237	35.2	9.9	54.9	64	>128
CLI			157	29	18	5	4	41	19	12	221				36.8	3.5	59.7	4	>16

PEN = Penicillin; AMP = Ampicillin; AMC = Amoxicillin/Clavulanate 2/1; EFT = Ceftiofur; ENR = Enrofloxacin; FFC = Florfenicol; TET = Tetracycline; STX = Trimethoprin/Sulfamethoxazole 1/19; TIA = Tiamulin; TIL = Tilmicosin; TUL = Tulathromycin; CLI = Clindamycin. S = Susceptible; I = Intermediate; R = Resistant; MIC = Minimal Inhibitory Concentration; The dilution ranges of individual antimicrobials are delimited in the grey zone. MIC values in the grey zone indicate MIC values higher than the highest concentration in the range. Values corresponding to the lowest concentration tested indicate MIC values less than or equal to the lowest concentration in the range. The MIC_50_ and MIC_90_ values represent the lowest concentration (mg/L) inhibiting the growth of 50% and 90% of the isolates in the bacterial culture with a density of 10^5^ CFU/mL.

**Table 2:** The origin of *S. suis* isolates.

Body Site of Isolation	Number of Isolates					
	2018	2019	2020	2021	2022	Total
nasal swabs	13	8	10	17	4	52
tonsils	-	9	-	-	-	9
lower respiratory tract	51	31	43	54	41	220
lymph nodes	11	16	17	19	14	77
joint	3	4	5	10	5	27
brain	9	12	13	26	8	68
digestive system	2	2	6	6	3	19
urogenital tract	2	1	2	2	1	8
skin	2	2	-	-	-	4
not specified	1	1	1	19	-	22

**Table 3:** The set for the antimicrobial susceptibility testing of *S. suis* with tested concentrations (mg/L) of antimicrobials, and the breakpoints used.

PEN	AMP	AMC *	EFT	ENR	FFC	CLI	TIA	TIL	TUL	TET	SXT **
4	4	32	16	8	64	16	32	128	128	32	PGC
2	2	16	8	4	32	8	16	64	64	16	4
1	1	8	4	2	16	4	8	32	32	8	2
0.5	0.5	4	2	1	8	2	4	16	16	4	1
0.25	0.25	2	1	0.5	4	1	2	8	8	2	0.5
0.125	0.125	1	0.5	0.25	2	0.5	1	4	4	1	0.25
0.06	0.06	0.5	0.25	0.125	1	0.25	0.5	2	2	0.5	0.125
0.03	0.03	0.25	0.125	0.06	0.5	0.125	0.25	1	1	0.25	0.06

PEN = Penicillin; AMP = Ampicillin; AMC = Amoxicillin/Clavulanate 2/1; EFT = Ceftiofur; ENR = Enrofloxacin; FFC = Florfenicol; CLI = Clindamycin; TIA = Tiamulin; TIL = Tilmicosin; TUL = Tulathromycin; TET = Tetracycline; STX = Trimethoprin/Sulfamethoxazole 1/19; PGC = Positive Growth Control. Susceptible; Intermediately resistant; Resistant, * Concentration in the table is valid for amoxicillin. ** Concentration in the table is valid for trimethoprim.

## Data Availability

The data are available on request from the corresponding authors. More detailed data about farms are not publicly available to protect the privacy of farm owners.
